# Long Duration Ultrasound Combined with Platelet-Rich Plasma Injection for Return to Sport after Soft Tissue Injury: A Single Center Study

**Published:** 2023-08-30

**Authors:** Jason Krystofiak, Jan Bruins, Ethan Bates, Josh Kummer

**Affiliations:** 1 Department of Sports Medicine, RWJ Barnabas Health, New Jersey, USA;; 2 Department of Athletic Rehabilitation, RWJ Barnabas Health, New Jersey, USA;; 3 Department of Biomedical Engineering, University of Cincinnati, Ohio, USA;

**Keywords:** Platelet rich plasma, Regenerative medicine, Return to sport, Sustained acoustic medicine, Long duration ultrasound, Sports medicine

## Abstract

**Objective:**

The use of Long-Duration Ultrasound (LDU) and Platelet-Rich Plasma (PRP) treatments to facilitate injury healing and pain relief are typically utilized independently in sports medicine. Our study aimed to investigate the combined regenerative effect of daily LDU with high-concentration single-injection PRP for treating sport-related musculoskeletal injuries.

**Methods:**

In total, thirty-five competitive athletes (n=35) with grade II sprains and strains and tendinopathies injured during sport and unable to continue to play were sequentially administered PRP (n=20, 20.4 yoa, 18 male, 2 female) or PRP+LDU (n=15, 20.27 yoa, 14 male, 1 female). In the PRP treatment group, each subject was treated with a single injection of PRP consisting of 1.28 billion platelets/mL under ultrasound-image guidance to the injury site. The PRP+LDU treatment group received the same PRP injection procedure with a 14-day, 4 hr per day, 18,720 J ultrasound treatment applied over the injection site. The Numeric Ration pain Scale (NRS, 0–10), Range of Motion (ROM, 0–100%), Clinical Strength numeric score (CS, 0–5), and time of injury to return to sport (days) were measured at baseline and Return to Play (RTP). The global health improvement score (GROC −7 to +7) was measured upon RTP.

**Results:**

All patients completed rehabilitation and returned to the sport after debilitating injuries. PRP+LDU returned athletes to play 21.33 days quicker (p<0.0001), decreased injury pain by 0.88 NRS points (p=0.0086), and improved patient global health by 1.28 points GROC (p<0.0001) over PRP treatment alone (95% Confidence interval, 11.26 to 31.40 days faster). There were no significant differences in strength (p=0.498) or range of motion (p=0.8581) improvement between PRP and PRP+LDU at the RTP or baseline patient demographic variables.

**Conclusion:**

Adding LDU at-home treatment to PRP injection therapy significantly reduces the time to return to sport, increases pain reduction, and improves overall health for patients recovering from sport-related injury. The daily LDU treatment facilitates and enhances regenerative medicine therapies such as PRP.

## INTRODUCTION

Musculoskeletal sprains and strains are the most common injuries among collegiate athletes and were found to account for 45.9% of all competition injuries and 52.1% of all injuries requiring more than 7 days of rest [1]. Between 1988 and 2004, 34.5% of reported college football injuries were sprains or strains [2]. Proper recovery is crucial for athletes to adapt and improve their performance. Competitive sport leads to chronic stress of performance and overtraining, potentially disrupting the body’s recovery processes, such as muscle repair and glycogen replenishment [3]. Injuries require adequate time for recovery and rehabilitation. If athletes do not allow their bodies to heal properly or return to activity early, they may exacerbate the injury or develop compensatory movement patterns that can induce fatigue, increasing the risk of further injuries and resulting in underperformance [4–6]. Adequate recovery is essential for the athletes’ optimal performance and timely return to play [7,8]. The athletes’ injuries and associated underperformance lead to significant economic costs. The National Collegiate Athletic Association (NCAA) estimated that the medical expenses for college athletes’ injuries can range from thousands to millions of dollars, depending on the severity and type of injury [9]. In professional sports, the economic cost of athlete injuries can be significant due to factors such as lost revenue from ticket sales, decreased viewership, and potential impact on sponsorship deals. Recent advancements in biomedical technology advancement have allowed combing medical devices and biological interventions leading to new, innovative, and more effective therapies to enhance the healing process and reduce the time the RTP.

Platelet-Rich Plasma (PRP) is a naturally derived biologic that can reduce pain and has the potential to reduce the return to activity time from musculoskeletal injuries [10,11]. The higher prevalence and severity among athletes significantly affect the ability to move and work [12]. Elite athletes regularly utilize PRP treatment to enhance healing from soft tissue injuries, reduce surgical interventions, and reduce the use of corticosteroids and Non-Steroidal Anti-Inflammatory Drugs (NSAIDs). The average concentration of platelets in whole blood is 200,000/mL [13]. The PRP is segregated from whole blood cells in differential centrifugation, separating Red Blood Cells (RBC) and platelet-containing plasma. In the second centrifugation stage, the plasma is segregated into Platelet-Rich Plasma (PRP) and Platelet-Poor Plasma (PPP). Finally, the PRP is utilized for clinical application [13–16].

Platelet-induced blood coagulation is integral to soft tissue healing [17]. A vascular injury activates platelets as they approach the injury site through integrin αIIbβ3 inside-out signaling. Inside-out signaling is facilitated by the interaction of Glyco-Protein (GP.) Ib-IX-V/collagen-v WF and/or GP-VI/collagen interactions, activating Src family kinases. This phosphorylates downstream intracellular proteins, including the PLCγ tyrosine and Phosphatidylinositol-3-Kinase (PI3K). The regulation of downstream Ca2+-dependent IP3 and Ca2+-independent Rap-1 pathways leads to platelet coagulation, aggregation, clot retraction, adhesion, and thrombus consolidation [18–20]. Platelets are also associated with released Growth Factors (GF) known as Platelet-Derived-Growth-Factors (PDGF), associated with (i) revascularization through endothelial cells migration, proliferation, differentiation, and stabilization into the blood vessels; (ii) fibroblast migration, proliferation, and activation to restore the damaged connective tissue; and (iii) the proliferation and differentiation mesenchymal stem cells into tissue-specific cells [21]. PDGF has been shown to stimulate osteoblasts to enhance fracture healing and tendon and ligament healing [21–23]. The GF, responsible for inflammation, transforms GF βI, which recruits fibroblasts to repair connective tissue and inflammatory cells [24]. As a result, platelets recruit many regenerative cells and require nutrients at the injury site to accelerate the healing process [21,23,24]. PRP has a platelet concentration 5 times higher than the physiological range of healthy human whole blood. This increased concentration enhances the physiological healing response, resulting in increased proliferation of regenerative cells such as osteoblasts, fibroblasts, and mesenchymal stem cells to the wound site [13].

Rossi et al. showed a significant decrease in RTP time of an average of 3.9 days in the healing of acute muscle tears in athletes [11]. The PRP injections are typically administered by ultrasound-guided injection into the injury site. Multiple clinical studies have shown the efficacy of ultrasound-guided injections in carpel tunnel syndrome and sacroiliac joint disability and pain [25,26]. A comprehensive meta-analysis by Belk et al. demonstrates that the PRP treatment significantly improves Western Ontario and McMaster Universities Osteoarthritis Index (WOMAC), Visual Analog Scale (VAS) for pain, and Subjective International Knee Documentation Committee (IKDC) scale relative to hyaluronic injection only treatment in Osteo Arthritis (OA) patients [27].

Long Duration Ultrasound (LDU) treatment is a method of stimulating the healing process through the mechanical properties of high-dosimetry continuous ultrasound waves [28]. LDU treatment is typically applied daily for 2 to 4 weeks by a wearable device to accelerate the natural progression of soft tissue injury repair. LDU is applied to tendon and ligament injuries, muscle strains, and osteoarthritis [28]. This therapy has been shown to be an effective, non-conflicting treatment while allowing patients to pursue other adjunct treatment methods, such as traditional physical therapy [29]. LDU is a treatment that the patient can undergo independently at home without an athletic trainer or medical professional’s assistance or the need to travel to a physician [30]. Each ultrasonic transducer produces approximately 10,000 joules of energy over four hours, resulting in approximately 20,000 joules delivered when two ultrasound transducers are used over four hours [31]. The ultrasound that is produced by the transducers causes the heating of deep tissues instead of epithelium, which causes vasodilation, increasing the blood flow to tissues and allowing enhanced oxygen and nutrient allocation to the damaged tissues [28,31]. This increases the body’s healing speed and reduces inflammation. LDU has been shown to aid in the repair of several biological tissues, including tendons, ligaments, muscle, bone, and tendon-bone junctions [32]. LDU has also been shown to increase the topical absorption of substances due to the increased permeability of membranes [33]. The increase in temperature of deep tissue increases the rate of chemical reactions, which are the basis for tissue repair.

Suh et al. have shown that enhanced platelet penetration using ultrasound therapy has been shown to augment stagnant decrease in depth of striae distensae from 0.75 mm to 0.27 mm after two months of treatment, demonstrating synergic effects of PRP and ultrasound therapy [19]. However, besides Suh et al., little clinical and scientific evidence demonstrates the combined effect of PRP and ultrasound treatment. LDU has been shown to reduce pain, increase Range of Motion (ROM), and increase the healing rate of tissue [29]. Eighteen (18) previously treated athletes who had little or no response to traditional therapies in muscle strains, tendinopathies, and tears were treated with LDU in conjunction with active treatments. After the addition of LDU, athletes experienced a reduction in pain, increased ROM, functionality, and eventual return to work/sports [29]. Best et al. have shown LDU efficacy in treating elbow and Achilles tendinopathy. After six weeks of treatment, subjects reported a mean 3.94-point decrease in pain on the 11-point Numerical Rating Scale (NRS) due to chronic tendinopathy of the elbow and ankle [34]. This study aims to evaluate the effectiveness of combined PRP+LDU in treating soft tissue injury, reducing pain, returning to activity, and overall patient health and satisfaction with the treatment.

## METHODOLOGY

### Study design

**Figure 1:**
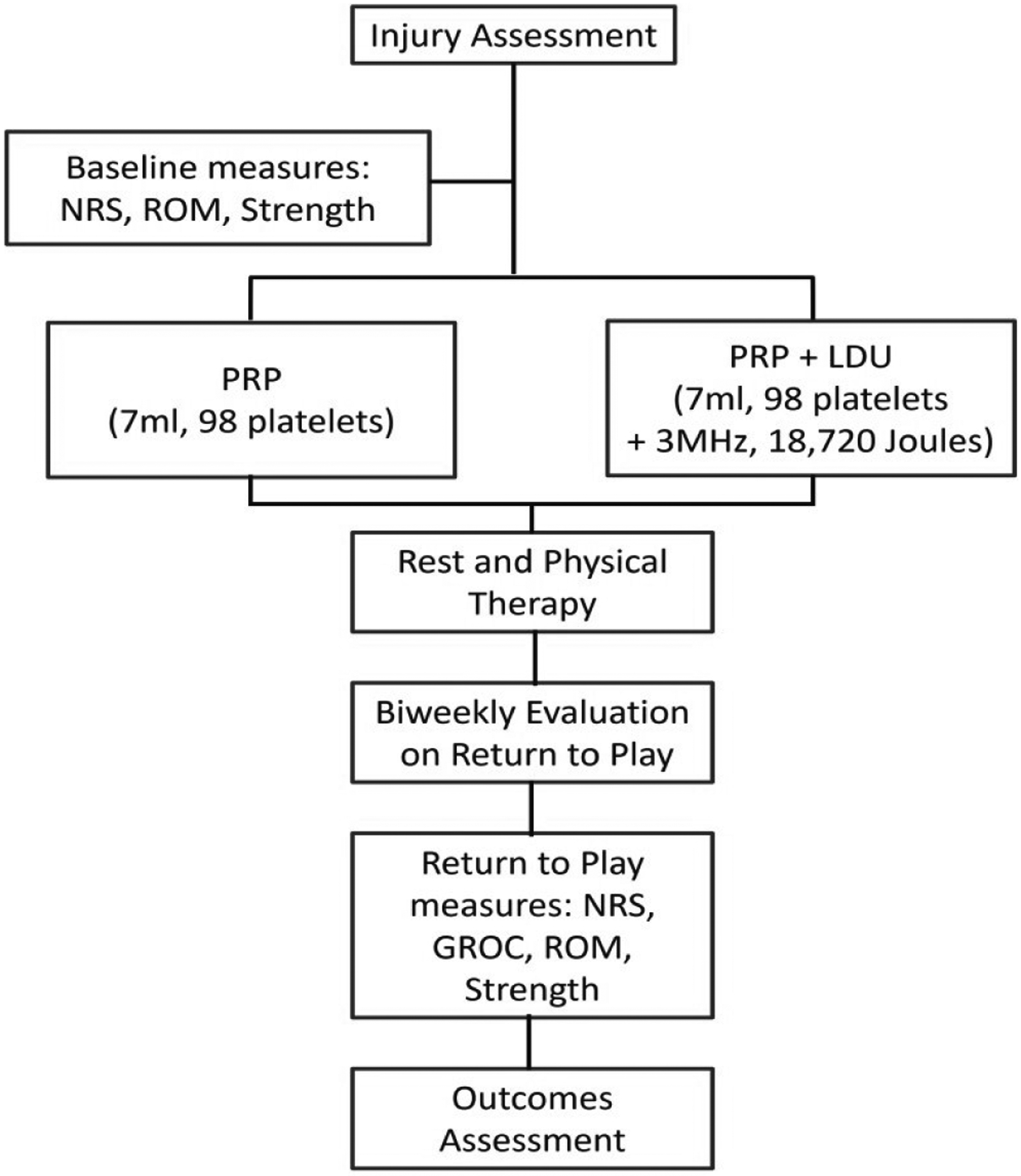
Study flowchart illustrating study design. **Note:** Numeric Ratting Scale (NRS); Range of Motion (ROM); Platelet Rich Plasma (PRP); Long Duration Ultrasound (LDU); and General Rate of Change (GROC).

This was a single-center, open-label; sequential treatment study conducted on elite athletes in the United States and adhered to the principles outlined in the Declaration of Helsinki. Thirty-five patients enrolled in the study during the 2022–2023 collegiate sports season. The patients were distributed sequentially into two groups, PRP and PRP+LDU, to prevent bias in athlete care. The study was designed to equally manage the care of the subjects, as illustrated in Figure 1. The subjects were functionally assessed clinically using the Medical Research Council (MRC) guidelines, where 0 is paralysis, and 5 is full muscle contraction against the examiner’s resistance [35]. The pain reduction Numeric Rating Scale (NRS, 0–10), global health improvement scale (GROC, −7 to +7), return to sport time (days, date of injury to return to sport), Range of Motion (ROM, 0–100%), and Clinical Strength numeric score (CS, 0–5) were used as outcomes pre/post-intervention. Team athletic trainers and a single school sports medicine physician made the final decision to return an athlete to play after the student-athlete demonstrated the ability to undergo competition-realistic exercises and achieve designated functional marks. All patient outcomes were measured in-season after an athlete/subject was eligible to return to activity after being treated post-injury. At 6 months patient follow-up for reinjury was conducted during physical health visit.

#### Patients or participants:

Eligible patients (n=35) included athletes over 18 with moderate musculoskeletal injuries, including grade 2 strains or sprains or established tendinopathies. These injuries had not shown improvement with conservative management for 1-week and were treated with PRP to augment healing and reduce RTP time. Exclusion criteria were patients with active infection, undergone prior surgery of the targeted area in six months, had local corticosteroid or PRP injection in the past three months, had allergy concerns or unwilling to use NSAIDs for more than two weeks. The LDU patients were well-trained to use the device before self-treatment. The data were collected between January 2022 to January 2023 under the supervision of an attending physician under an approved IRB (Advarra I.R.B., September 2021).

Patients were treated as needed, with 94.1% being male due to the higher injury rate of football. The test (PRP+LDU, n=15 which include 14 males, 1 female) and control (P.R.P., n=20, 18 males, 2 females) groups had a similar sex ratio and were not confounding variables. All athletes were treated with the appropriate standard of care intervention.

#### Procedures:

PRP was prepared according to the manufacturer’s instructions (PurePRP^®^, EmCyte Corporation). The 60 mL PRP preparation kit was used to treat large injury areas, where 6 mL of anticoagulant sodium citrate was first drawn into a 60 mL syringe using a filter needle. Under sterile conditions, 54 mL of whole blood was drawn from an antecubital vein of the patient into the syringe. The blood and anticoagulant were then gently mixed to avoid coagulation. The resulting solution was then centrifuged and processed, and 7 mL of PRP was obtained. The 30 mL PRP preparation kit was used for smaller injury areas, and 4 mL of PRP was obtained. This PRP collection system provides an average of 1.29 billion platelets ± 362 million per mL of PRP [36]. The prepared PRP solution was then injected into the injured tissue with ultrasound guidance. The amount of PRP injected depended on the injury’s size and location. High ankle anterior tibiofibular sprains were treated with 1–2 mL, patellar tendinopathies were treated with 2–4 mL, and hamstring/quadriceps strains were treated with 7 mL of PRP solution. This variation of injection amount is consistent with the 2–6 mL range outlined in Orlandi et al. for different injury types [36,37].

Proper needle location was confirmed through direct ultrasound visualization of the injectate. After injection, 15 of the 35 athletes were given an LDU. Treatment system (SAM^®^ 2.0, Sustained Acoustic Medicine, ZetrOZ Systems Corporation) to be utilized post-PRP. Athletes were prescribed to use LDU directly over the injection site for four hours a day with both applicators for a total of 18,720 joules delivered. LDU treatment was administered for 14 days after the PRP injection with team medical staff following up daily with each patient to help facilitate LDU treatment.

### Statistical analyses

The primary outcome measure was RTP time. However, the study was powered by pain reduction due to the prevalence of existing literature on pain. Secondary outcome measures included global health improvement, pain reduction, muscle strength, and range of motion. The student’s t-test (t test) was used for all endpoints to determine significance. All endpoints were dependent on the presence of LDU treatment. There were two subgroup analyses done, which considered endpoints by injury type. These were also t-tests, with the active and control groups broken down into tendinopathies and sprains and strains for more specific analysis. Statistical significance was set for p ≤ 0.05 for all outcome measures.

## RESULTS

The PRP (n=20) and PRP+LDU (n=15) groups had an average age of 20.40 ± 1.31 and 20.27 ± 1.45 years, respectively (p=0.7805), as shown in Table 1. No initial patients were disqualified during treatment, despite one having initial non-compliance with PRP follow-up. Two subgroup analyses were done based on injury type.

**Figure 2:**
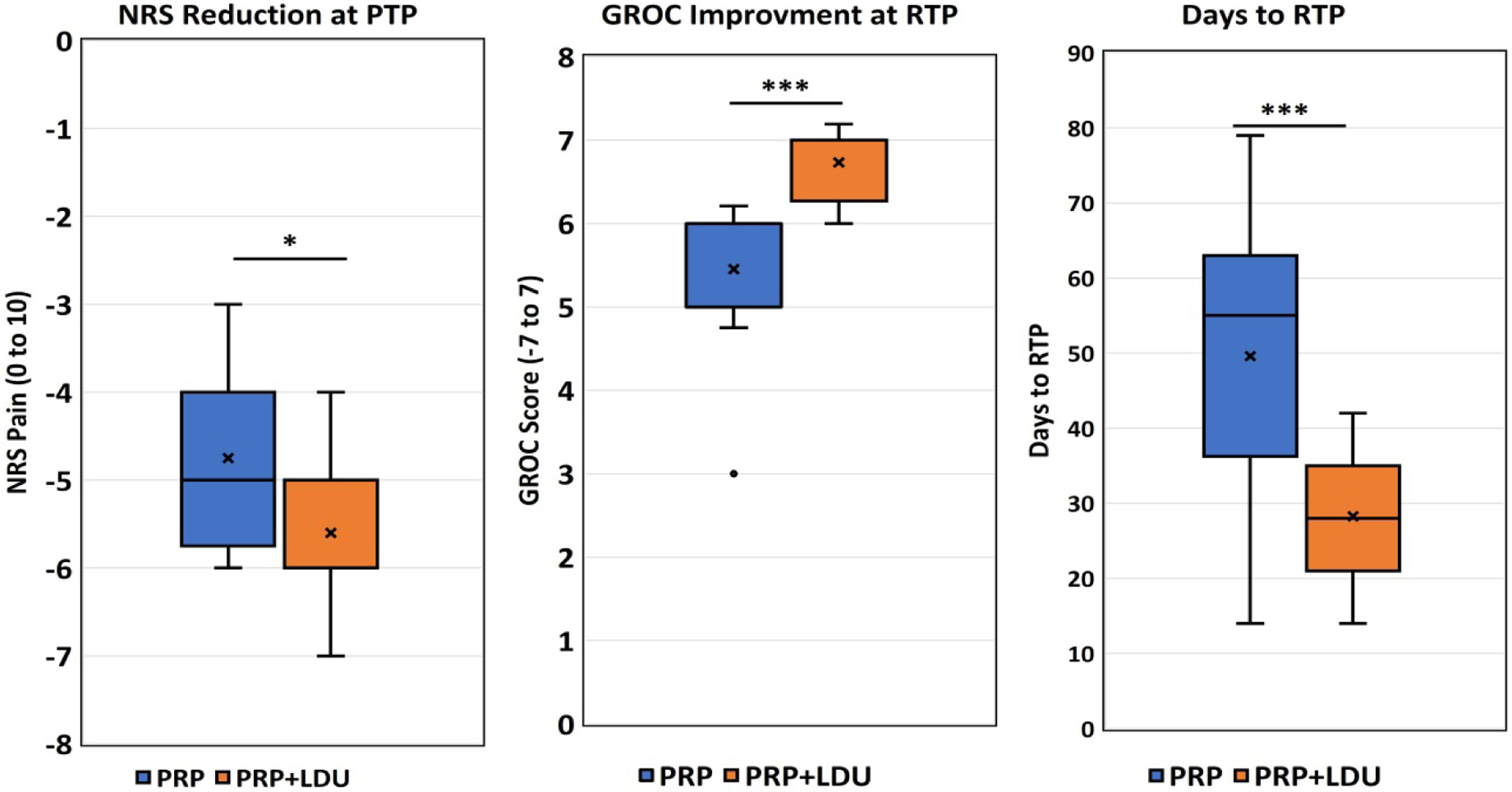
Return To Play (RTP) analysis. a) Pain reduction on NRS scale (0: No reduction, 10: No pain at RTP); b) Pain reduction on GROC scale (0: No improvement, 7: Complete improvement); c) Impact on the reduction of RTP duration during the reason. *P=<0.05, ***P<0.0001. **Note:** (■) PRP; (■) PRP+LDU.

The PRP+LDU-treated patients showed significant improvement in NRS, GROC, and RTP time over PRP-treated patients (Figure 2). The greatest improvement was recorded in the return of play time, with PRP+LDU treatment having a 21.33-day shorter RTP time than the PRP treatment, as seen in Table 2 (p=0.0001). In addition, the PRP+LDU group had a 0.88-point greater decrease in pain (p=0.0086) and a 1.28-point higher GROC score (p=0.0001). Strength change and ROM change were both statistically insignificant between treatment groups (p=0.4980, p=0.8581).

The subgroup analyses were conducted on pain and GROC outcomes. Pain reduction (p=0.0123) and GROC scores (p=0.0001) showed a significant improvement in PRP+LDU-treated musculoskeletal sprains and strains (n=26). As shown in Figure 2.

The RTP subgroup analysis showed a statistically significant 20.83 day decrease (p=0.0015) in RTP days in musculoskeletal strain and sprains (n=26) for the PRP+LDU treated group. As shown in Figure 2 and Table 3.

The days for RTP, along with athlete type, pain reduction, and GROC improvement, is tabulated in Table 3. Grade 2 hamstring strains and grade 2 anterior tibiofibula strains for football athletes were the most common play-stopping strains injuries treated during the study which responded well to both PRP and PRP+LDU treatment. Patella tendinopathy was the most common tendon condition treated which also responded well to both interventions. Athletes treated with PRP+LDU showed significantly faster RTP versus PRP alone within the same sport season. No adverse events were reported during or after treatment, and at 6-months there was no reported re-injury in either treatment group.

## DISCUSSION

A safe, predictable, and expedited RTP is critical for an elite athlete after a significant injury or chronic musculoskeletal condition. Musculoskeletal sports are common in physical sports leading to a significant amount of lost in-season play time, advertisement, and audience revenues to the team annually. Early return without compromising long time health has significant socioeconomic benefits. The utilization of athletic training-directed modalities is standard amongst collegiate and professional organizations. LDU is commonly used on athletes for chronic and acute pain relief and is readily available.

This study compares athlete outcomes at their RTP time. The PRP+LDU-treated subjects reached their outcomes 21.3 days faster than those who received PRP alone. In the performance-driven institution of collegiate athletics, an athlete side-lined with an injury can have a significant financial impact. A protracted RTP can have deleterious effects on the athlete’s physical and behavioural health, resulting in poor academic performance, affecting future athletic opportunities, and negatively impacting their team. Thus, reducing downtime while ensuring players are fully capable of performing is vital. The tight standard deviation range of PRP+LDU, when compared to PRP, indicates more consistent and predictable treatment and RTP time. A wide range of potential outcomes can make it difficult for athletic trainers to accurately predicate and communicate the availability of players to the coaching staff. The lack of variability in the PRP+LDU group reduces the subjectivity in the MRC scale and improves an athlete’s confidence in RTP. No athletes reinjured the treated area within six months of follow-up. Kilcoyne et al. reported a 6.2% reinjury rate in grade I and II hamstring strains. There was no statistical difference in RTP between initial and secondary injury [38]. The lack of reinjury in this study demonstrates not only faster healing but also healing that reduces the risk of re-injury.

While no significant change was observed in strength and ROM at RTP, the PRP+LDU treatment significantly reduces the number of RTP days, which is correlated to the strength and ROM of the patient. There may be a minor detection bias in measuring pain reduction and GROC score, as in those who received LDU treatment may exaggerate symptom improvement.

No adverse events were identified with the addition of LDU, and there was no impact on treatment durability at 6-months for athletic patients. This study did not explore mechanistic reasons why the PRP+LDU group returned to play faster than the PRP group. LDU is acoustic, mechanical wave propagation through medium providing essential mechanical force to enhance platelets penetration and delivery of PDGF. Thus, platelets induce enhanced cellular and molecular activities, including migration, proliferation, and regeneration [21,23,24]. More in-depth studies would be required to review the possible mechanism in future studies. This study has some limitations, including a smaller sample size in the PRP+LDU group and limited female athlete representation, and no follow-up imaging (ultrasonography, etc.) after RTP. Future research could radio graphically measure healing progression during RTP and include a higher-powered study to further evaluate the use of PRP+LDU in soft tissue-related injuries in the general population.

## CONCLUSION

Our findings suggest that patients being treated with PRP therapy for healing a soft tissue injury may receive additional benefits with the addition of 14-day LDU therapy. The combination of PRP+LDU therapy provides a significant reduction in time to return to sport without impacting the long term durability of the soft tissue. It increases pain reduction and overall health improvement for patients recovering from a sport-related injury. Non-invasive LDU therapy provides a valuable tool to expedite tissue regeneration and should be considered by practitioners to augment regenerative medicine approaches such as PRP.

## Figures and Tables

**Table 1:** Patient demographic information, Body Mass Index (BMI), Standard Deviation (SD).

Subject type	Mean age ± SD.	Gender	Mean BMI ± SD.	Mean baseline injury pain ± SD.	Treatment time ± SD
**PRP**	20.40 ± 1.31	18-Male, 2-Female	25.75 ± 2.77	5.13 ± 0.83	49.6 ± 17.2
**PRP+LDU**	20.27 ± 1.45	14-Male, 1-Female	28.04 ± 5.49	5.6 ± 0.83	28.0 ± 8.10
**p value**	0.7805	-	0.1961	0.1103	0.0001

**Table 2:** Patient outcomes at Return to Play (RTP), Platelet-Rich Plasma (PRP), and Long-Duration Ultrasound (LDU).

Outcome measure	PRP Mean ± PRP mean ± SD S	PRP+LDU Mean ± PRP+LDU mean ± SD S	Mean group difference and confidence interval	p value
**Pain reductionB (NRS)**	4.65 ± 0.93	5.53 ± 0.92	0.88 (0.24 to 1.53)	0.0086
**Global health improvement (GROC)**	5.45 ± 0.76	6.73 ± 0.46	1.28 (1.73 to 0.83)	0.0001
**Injury to return to sport (Days)**	49.60 ± 17.67	28.27 ± 8.38	21.33 (11.26 to 31.40)	0.0001
**Strength change on return to sport**	22.0% ± 14.4%	25.3% ± 14.1%	3.30%(−6.6% to 13.2%)	0.498
**Range of motion change on return to sport (% ROM)**	21.5% ± 14.6%	20.7% ± 14.1%	−0.83% (−8.57% to 10.2%)	0.8581

**Table 3:** Key Patient Outcomes at Return to Play (RTP) by condition, location and athlete type, Platelet-Rich Plasma (PRP), Long Duration Ultrasound (LDU), Return to play (RTP).

Treatment	Injury type and PRP injection site	Sport	Gender	BMI	NRS pain reduction at RTP	GROC at RTP	Days from injury to RTP
PRP	Grade 2 hamstring strain	Football	M	28.89	−6	6	64
	Grade 2 hamstring strain	Football	M	29.84	−6	6	22
	Grade 2 hamstring strain	Football	M	24.8	−4	6	40
	Grade 2 hamstring strain	Football	M	25.76	−6	5	37
	Grade 2 hamstring strain	Football	M	21.49	−6	5	36
	Grade 2 quad strain	Football	M	28.99	−5	6	54
	Grade 2 quad strain	Football	M	25.97	−4	6	79
	Grade 2 quad strain	Football	M	24.27	−5	5	59
	Grade 2 wrist sprain	Football	M	27.53	−5	5	14
	Patellar tendinopathy	Football	M	29.73	−3	3	30
	Patellar tendinopathy	Football	M	22.73	−5	6	49
	Patellar tendinopathy	Football	M	24.4	−4	5	63
	Grade 2 hamstring strain	Track	M	22.68	−5	5	56
	Grade 2 hamstring strain	Track	M	22.68	−5	6	70
	Grade 2 hamstring strain	Track	M	23.55	−4	6	56
	Grade 2 hamstring strain	Track	M	23.1	−5	5	56
	Grade 2 hamstring strain	Track	M	22.97	−6	6	49
	Grade 2 hamstring strain	Track	M	26.02	−4	6	63
	Patellar tendinopathy	Track	F	26.48	−3	5	25
	Patellar tendinopathy	Track	F	21.81	−4	6	70
**Mean**				**25.18**	**−4.75**	**5.45**	**49.6**
**SD**				**2.68**	**0.97**	**0.76**	**17.69**
PRP+LDU	Grade 2 hamstring strain	Football	M	20.54	−6	6	42
	Grade 2 hamstring strain	Football	M	28.5	−6	7	35
	Grade 2 anterior tib-fib sprain	Football	M	35.43	−7	7	21
	Grade 2 anterior tib-fib sprain	Football	M	26.97	−5	7	19
	Grade 2 anterior tib-fib sprain	Football	M	35.63	−6	6	21
	Grade 2 anterior tib-fib sprain	Football	M	34.14	−6	6	28
	Grade 2 anterior tib-fib sprain	Football	M	34.98	−6	7	14
	Grade 2 MCL sprain	Football	M	25.1	−7	7	30
	Patellar tendinopathy	Football	M	28.5	−4	6	24
	Patellar tendinopathy	Football	M	23.01	−7	7	21
	Patellar tendinopathy	Football	M	34.14	−5	7	28
	Grade 2 quad strain	Soccer	F	21.9	−5	7	35
	Grade 2 hamstring strain	Track	M	22.71	−5	7	35
	Grade 2 hamstring strain	Track	M	23.1	−5	7	42
	Quad tendinopathy	Track	M	25.9	−4	7	29
**Mean**				**28.04**	**−5.6**	**6.73**	**28.27**
**SD**				**5.49**	**0.99**	**0.46**	**8.38**
**P value**				**0.0794**	**0.0163**	**<0.0001**	**<0.0001**
